# Association Between Residual Pericervical and Apical Dentine and Vertical Root Fracture in Endodontically Treated Molars: A Case‐Control Study

**DOI:** 10.1002/cre2.70293

**Published:** 2026-02-03

**Authors:** Kwangsoon Lee, Shanon Patel, Manjeet Ahlowalia, Ruth Perez Alfayate, Federico Foschi

**Affiliations:** ^1^ Practice Limited to Endodontics Tokyo Japan; ^2^ Department of Endodontology King's College London Dental Institute London UK; ^3^ Department of Endodontics Universidad Europea de Madrid Madrid Spain; ^4^ Department of Endodontology UCL Eastman Dental Institute London UK

**Keywords:** apical dentin, diagnosis, molars, pericervical dentin, risk factors, root canal treatment, vertical root fracture (VRF)

## Abstract

**Objective:**

Vertical root fracture (VRF) in endodontically treated molars (ETMs) is a multifactorial condition. However, the relationship between residual pericervical and apical dentine in ETMs and VRF has yet to be fully assessed. This study aimed to investigate the association between residual pericervical and apical dentine and VRF in ETMs.

**Material and Methods:**

ETMs with VRFs (44 cases) and those without VRFs (92 controls) were included. Residual dentine at pericervical level and apical terminus of root canal filling (RCF) were assessed based on the ratio between the mesiodistal widths of the RCF and root on periapical radiographs. The ratio was converted into four categories: “intact canal”, “minimum preparation”, “traditional preparation”, and “excessive preparation” based on calculated cut‐off values. History of root canal re‐treatment (reRCT), and time from the primary root canal treatment (pRCT) were assessed as cumulative factors. Descriptive and logistic regression analyses were used to identify risk factors for VRF. Receiver operating characteristic curves were constructed, and the corresponding area under the curve (AUC) was used to determine the model as a diagnostic tool.

**Results:**

“Excessive” category at both pericervical and apical dentine was more frequently observed in teeth with VRF (81.8%; 36/44, 61.4%; 27/44) than in the control group (65.2%; 60/92, 10.9%; 10/92). Residual apical dentine, tooth type, history of reRCT, and time from pRCT ≥ 15 years were significantly associated with VRF in the multiple binary logistic regression analyses (*p* < 0.05).

**Conclusions:**

Successful pRCT with minimum canal preparation, particularly at the apical level, is essential to minimize the likelihood of VRF. In ETMs with an isolated periodontal probing depth ≥ 5 mm, assessing residual apical dentine, tooth type, reRCT history, and time since pRCT can effectively differentiate VRF from non‐VRF teeth (AUC, 0.940; *p* < 0.001), offering valuable diagnostic guidance.

## 1 Introduction

Vertical root fracture (VRF) is a major cause of tooth loss, particularly in endodontically treated teeth (ETT) (Lee et al. 2023; Axelsson et al. 2004), and its diagnosis remains challenging due to multifactorial etiology and overlapping clinical presentations (Patel et al. 2022). The etiology of VRF can be divided into predisposing and contributory factors. Predisposing factors include tooth location, root canal anatomy/morphology, occlusal loading factors, parafunction, the structural integrity of the tooth, the biomechanical property of dentine, and diet (Patel et al. 2022). Endodontic and restorative procedures, such as post placement (Lee et al. 2023; Kishen et al. 2004; Maddalone et al. 2018) and condensation during obturation (Lertchirakarn et al. 2003; Sathorn et al. 2005), and root dentine loss (Ikram et al. 2009; Ossareh et al. 2018), are contributory factors. VRF most frequently occurs in the premolars and mandibular first molars (Lee et al. 2023; Chan et al. 1999; Yoshino et al. 2015), owing to their mesiodistally narrow, concave, or curved root morphologies (Lertchirakarn et al. 2003; Sathorn et al. 2005; Chan et al. 1999). These roots tend to be subjected to greater stress from endodontic procedures and occlusal loading (Kim et al. 2010; Li et al. 2015; Yoon et al. 2018). Typically, a narrow, isolated periodontal pocket has been associated with VRF (Pitts and Natkin 1983; PradeepKumar et al. 2016). A study assessed 228 teeth with an isolated probing depth (PD) ≥ 5 mm revealed that isolated periodontal pockets in molars were also associated with other pathological conditions such as apical periodontitis (AP) due to endodontic treatment failure, while those in premolars were exclusively associated with VRF (Lee et al. 2023). Furthermore, ETT restored with endodontic posts were significantly associated with VRF (Lee et al. 2023); however, molars usually do not require posts, when necessary, the distal or palatal roots are more suitable (Guldener et al. 2017). Nevertheless, the mesial roots are more susceptible to VRF (Guldener et al. 2017). Thus, other factors may contribute to VRF development in endodontically treated molars (ETMs). Root canal preparation removes root dentine from pericervical (roughly 4 mm above and apical to crestal bone) (Clark and Khademi 2010) to apical area (normally at the level of −0.5 to −1 mm from the root apex). However, the effect of root dentine loss on VRF remains unclear because clinical studies on the topic are scarce. The aim of this study was to investigate the association between residual pericervical and apical dentine and the occurrence of VRF in ETMs. In addition, history of root canal re‐treatment (reRCT) and time elapsed from primary root canal treatment (pRCT) were assessed to investigate cumulative factors and the effect of fatigue.

## 2 Materials and Methods

This case‐control study compared ETMs with VRF (cases) with those without VRF (controls). The study design followed the Strengthening the Reporting of Observational Studies in Epidemiology (STROBE) guidelines for reporting observational studies (von Elm et al. 2008). Ethical approval for this study was granted by the BDM Research Ethics Office of King's College London (LRU‐20/21‐21498A). Verbal and written informed consent was obtained from all participants. A prior power analysis was conducted. Silva et al. (2021) reported post rates of 32% and 53% in the control and VRF groups, respectively. This result indicates that presence of post is a negative factor increasing probability of VRF (odds ratio (OR) = 2.4; *p* = 0.007). Based on this effect size, it was determined that a sample size of 136 teeth is required to achieve 80% power and 95% confidence using a logistic regression model.

### 2.1 Sample Selection

**Figure 1:**
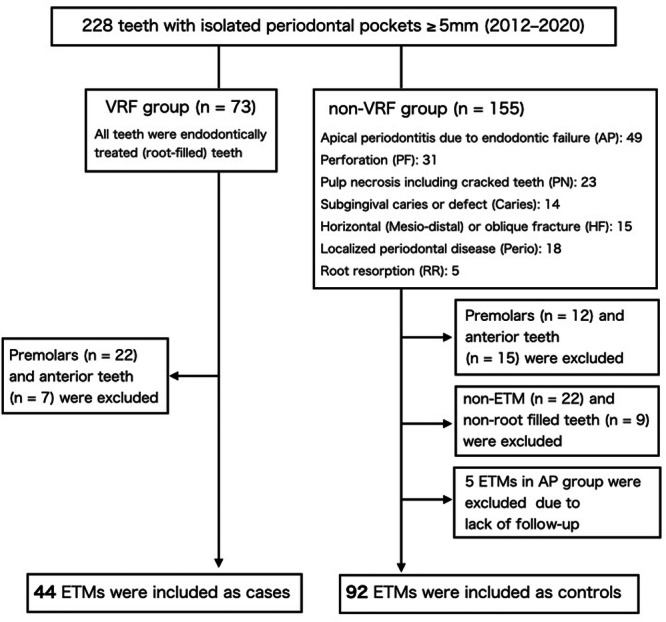
Flow chart showing the enrollment process.

A total of 136 ETMs (44 cases vs. 92 controls) were selected from our previous observational study including 228 teeth with confirmed isolated PD ≥ 5 mm from 221 patients who visited a specialist endodontic clinic in Tokyo between 2012 and 2020 for assessment or treatment (Lee et al. 2023). An isolated periodontal pocket was defined as “a localized periodontal defect with normal sulcus depth on either side” (Lee et al. 2023). The 228 teeth were initially classified into eight groups based on the most possible causes of the PD measuring ≥ 5 mm: (1) VRF, (2) subgingival caries, (3) horizontal (mesio‐distal) or oblique fracture, (4) localized periodontal disease, cemental tear, (5) perforation, (6) root resorption, (7) pulp necrosis including cracked teeth, and (8) unclear origin that could not be ascribed to groups (1)–(7), with AP due to endodontic failure (AP) (Figure 1). From the VRF group, root‐filled molars were selected as cases (*n* = 44). Controls (non‐VRF) were matched based on tooth type (molars) and endodontic treatment status (root‐filled teeth) (*n* = 92). Anterior teeth, premolars, and non‐ETT were excluded. Non‐root filled teeth, such as those in which pRCT was initiated but not completed, were also excluded. Teeth without follow‐up (*n* = 5) were excluded due to the potential for undetected VRF contributing to their PD ≥ 5 mm, leaving only teeth with confirmed healing. The inclusion and exclusion criteria are presented in Figure 1.

### 2.2 Diagnosis and Procedures

VRF was defined as “a complete or incomplete fracture initiated from the root at any level, usually directed buccolingually” (Rivera and Walton 2008). All procedures were performed by the first author under magnification using a dental microscope (OPMI PROergo, Carl Zeiss Meditec AG, Japan) which had a digital camera (Sony α7s, Tokyo, Japan) attached to it. Diagnosis of each condition was made visually during the treatment by confirming matching site of PD and each condition, osseous defect on cone‐beam computed tomography (CBCT) scans, and direct observation after tooth extraction. All VRFs were visually confirmed. For controls, reRCT was carried out under the rubber dam isolation.

### 2.3 The Outcome Assessment

The treatment outcome for controls was assessed by the author (KL) initially at the follow‐up based on the absence of symptoms, and absence (complete healing) or diminished size of the apical radiolucency (healing). A blinded examiner (KU), with 15 years of experience as a general dental practitioner and 5 years of experience as an endodontist, confirmed the outcome radiographically.

### 2.4 Patients' Data

Anonymized patient data included age; sex; medical history; dental history; signs and symptoms; and the depth, broadness, and location of periodontal pockets. Pre‐ and postoperative (only for controls) periapical radiographs (PAs) were obtained using MAX‐DC70 (70 kV, 4 mA. J. Morita MFG Corp, Kyoto, Japan), photostimulable phosphor plates, and a positioning device (CID‐4, Hanshin Technical Laboratory Ltd., Japan). Radiographs obtained between 2012 and August 2015 and those obtained after August 2015 were scanned using an i‐VIEW scanning system (J. Morita MFG Corp, Kyoto, Japan) and a VistaScan Mini View scanner (Dürr Dental SE, Beitigheim, Germany), respectively. Preoperative CBCT scans (67 out of the 83 teeth) were taken using the CS81003D (Carestream Dental, Atlanta, GA, USA), with the following protocol: voxel size, 75 μm; field of view (FOV), 5×5 cm; 5 mA; and 60 kV.

### 2.5 Variables

Age, sex, tooth type, proximal contacts, restoration type, presence of a post, the apical terminus of the root canal filling (RCF), residual pericervical and apical dentine, history of reRCT, and the time from pRCT were assessed.

### 2.6 Assessment of the Apical Terminus of RCF

The apical terminus of the RCF was assessed based on the radiographic apex and categorized as follows:

- **Short:** RCF shorter than the apical one‐third from the radiographic apex.
- **Adequate:** RCF ends within the apical one‐third but does not reach the radiographic apex.
- **Exact:** RCF reaches the radiographic apex.
- **Over:** RCF extends beyond the radiographic apex.

Two experienced endodontists (each with over 20 years of clinical experience) independently evaluated the apical terminus. Calibration was performed prior to the measurement using 10 randomly selected ETMs which were not part of the main study. These cases were re‐assessed 2 months later to confirm intra‐examiner agreement. In case of no agreement, final decision was made by the author. Cohen's kappa coefficients and 95% confidence intervals (CIs) were calculated to assess both intra‐ and inter‐observer agreement on the assessment.

### 2.7 Assessment of Residual Pericervical and Apical Dentine

#### 2.7.1 Measurement

**Figure 2:**
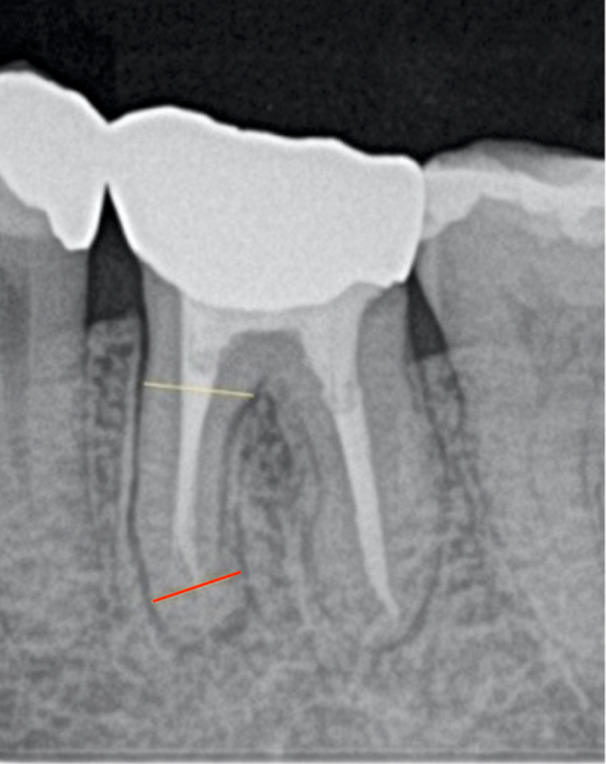
Reference points for measuring residual pericervical (furcation level) and apical dentine (apical terminus of theRCF).

Residual pericervical and apical dentine was assessed by calculating the ratio between the mesiodistal width of the RCF or post and the root width on PAs. The measurement was carried out at the coronal and apical references perpendicular to the root. Reference points were established at the furcation level and apical terminus of the RCF for the pericervical (Clark and Khademi 2010) and apical dentine assessment, respectively (Figure 2). When the RCF reached or exceeded the radiographic apex, the measurement was performed −1 mm above the radiographic apex. In teeth where RCF did not reach apical third of the root, the root canal (RC) width at the corresponding root level was measured. In cases where measurement was unfeasible due to canal obliteration, the mean RC‐to‐root width ratio of intact canals, used as a reference, was applied (Table 1).

In the case group, only roots with VRFs were assessed. In the control group, roots with a PD ≥ 5 mm were evaluated. All measurements were performed by the first author. Calibration was performed prior to the measurement using 10 randomly selected ETMs which were not part of the main study. A second measurement was performed 2 months after the initial measurement after masking previous data to confirm intra‐examiner agreement. In case of no agreement, decision was made by a third measurement. The obtained ratio was classified into four groups based on cut‐off values (Table 1).

#### 2.7.2 Categorization

The obtained ratios were categorized into four groups:

1. **Intact Canal**
2. **Minimum Preparation**
3. **Traditional Preparation**
4. **Excessive Preparation**

To determine cut‐off values, randomly selected 48 roots that were not part of the main study were evaluated. These included 24 roots from 10 ETMs with known instrument sizes and 24 roots from 10 intact molars. Both groups had the same tooth distribution: 4 mandibular first molars, 2 mandibular second molars, 2 maxillary first molars, and 2 maxillary second molars. The intact molar group had a mean patient age of 44 years (range, 27–61 years). The mean RC or RCF‐to‐root width ratios (RC or RCF/root) were calculated and used to define the threshold values for each category (Table 1).

Instrumentation guidelines were as follows:

- **Minimum Preparation**: #25/.04 or #25/.08
- **Traditional Preparation**: #30 or #40 with 0.04 or 0.06 taper
- **Excessive Preparation**: Any ratio exceeding the threshold for traditional preparation

**Table 1:** Cut‐off values for categorization of RC or RCF‐to‐root width ratios (RC or RCF/root).

	Pericervical	Apical
Intact canal	0.13 (± 0.03)	0.11 (± 0.04)
Minimum	0.23 (± 0.03)	0.16 (± 0.04)
Traditional	0.27 (± 0.01)	0.21 (± 0.04)
Excessive	> 0.27	> 0.21

### 2.8 History of Previous reRCTs and Time Elapsed From pRCT

The number of reRCTs, and the time from pRCT were obtained from clinical records and the patient's history. Teeth lacking this information were excluded. The final sample sizes were 120 (39 cases and 81 control) for reRCT frequency and 111 (35 cases and 76 controls) for time elapsed from pRCT.

### 2.9 Statistical Analyses

**Figure 3:**
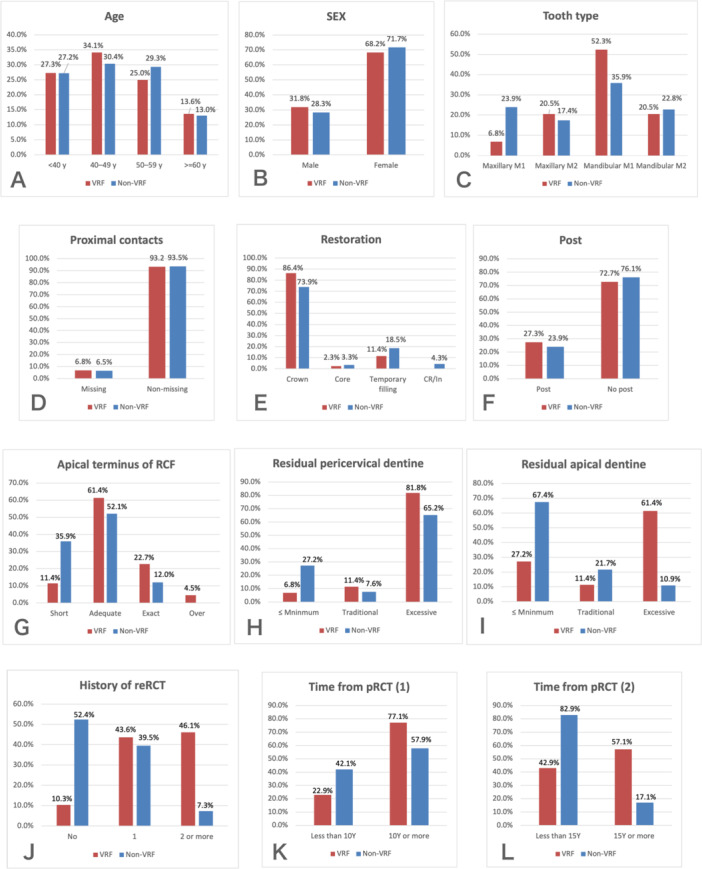
Distribution of teeth with VRF and non‐VRF according to the 12 variables (A–L). M1, first molar; M2, second molar; RCF, root canal filling; reRCT, root canal re‐treatment; pRCT, primary root canal treatment.

Statistical analyses were performed using SPSS software (version 15.0; SPSS Inc., Chicago, IL, USA). Descriptive analyses of the samples were carried out for the twelve factors differentiated by VRF versus non‐VRF (Figure 3). Non‐adjusted odds ratio (ORs) with 95% CIs for the association between independent variables and VRFs were initially obtained using simple binary logistic regression. Variables with *p* < 0.01 were selected for multiple binary regression analysis using the stepwise method. The adjusted OR was calculated, receiver operating characteristic (ROC) curves were constructed, and area under the curve (AUC), indexes, and rates of diagnostic and predictive validity were calculated to verify the reliability of the model as a predictive tool. Statistical significance was set at 5% (*α* = 0.05).

## 3 Results

The sample consisted of 136 ETMs (44 cases and 92 controls) from 132 patients (37 males and 95 females; mean age, 46.7 ± 9.9 years; range, 24–73 years). The majority of teeth were crowned (77.9%; 106/136) and four teeth had core only (crown removed). Five in cases and 17 in controls had temporary filling as they had an emergency treatment at general dental practices before visiting the author's practice. The frequency distributions of the patients’ demographic information and observed independent variables in both cases and controls are presented in Figure 3 and Supplimentary tables (T1–T12): S1. In both cases and controls, the mandibular first molars were the most frequent teeth (52.3%; 23/44, 35.9%; 33/92); the majority of teeth did not have a post (72.7%; 32/44, 76.1%; 70/92) (Figure 3C,F). Among teeth with posts, metal posts were prevalent in both VRF group and controls (50.0%; 6/12, 59.1%; 13/22), followed by screw posts (16.7%; 2/12, 40.9%; 9/22). Fiber posts were the least common (33.3%; 4/12, 0.0%; 0/22). VRF was prevalent in the mesial roots (61.4%; 27/44). Most cases had “adequate” apical terminus (61.4%; 27/44), and teeth rated as “over” was the least common (4.5%; 2/44) (Figure 3G). The mean RC or RCF/root for pericervical and apical dentine in the 48 roots used to calculate the cut‐off values are shown in Table 1, and those for cases and controls are shown in Table 2. Within the cases, “excessive” preparation was observed in 81.8% (36/44) (pericervical) and 61.4% (27/44) (apical) of teeth, respectively, this compared to “excessive” pericervical and apical preparations in 65.2% (60/92) and 10.9% (10/92) of controls, respectively (Figure 3H,I).

**Table 2:** Mean RC or RCF‐to‐root width ratios (RC or RCF/root) in cases and controls.

	Pericervical	Apical
Cases (VRF)	0.37 (± 0.11)	0.25 (± 0.12)
Controls (non‐VRF)	0.29 (± 0.10)	0.14 (± 0.06)

The mean RCF/root ratio in cases was significantly greater than that in the “traditional preparation” group (0.37 vs. 0.27), i.e. there was greater dentine loss in cases at both pericervical and apical levels compared to that in controls (Tables 1 and 2). Most controls maintained apical root dentine volume (67.4%; 62/92 was rated as ≤ “minimum preparation”) (Figure 3I). About 65% of controls (60/92) were rated as “excessive preparation” in pericervical dentine; however, unlike the mean ratio in cases (0.37), the mean ratio in controls was close to that in the “traditional preparation” group (0.29 vs. 0.27) (Tables 1 and 2). A history of reRCT was reported in 89.7% (35/39) of VRF group and 46.9% (38/81) of controls, and 46.1% (18/39) and 7.4% (6/81) had a history of two or more reRCTs, respectively (Figure 3J). pRCT was performed < 15 years prior in 42.9% (15/35) and 82.9% (63/76) of VRF group and controls, respectively (Figure 3L).

### 3.1 Simple Binary Logistic Regression Analysis

Tooth type (mandibular first molar), apical terminus of RCF, residual apical dentine, history of reRCTs, and time from pRCT ≥ 15 years were significant predictors of VRF (*p* < 0.05) (Table 3).

**Table 3:** Simple binary logistic regression analysis for probability of VRF. Raw odds ratio (OR) and 95% confidence interval (CI) by independent factors.

Variables	OR	95% CI	*p* value
Age	1.01	0.96–1.06	0.650
< 40 year	1 (ref.)		0.461
40–49 year	1.12	0.44–2.83	0.817
50–59 year	0.85	0.32–2.27	0.744
≥ 60 year	1.04	0.32–3.45	0.947
Sex			
Male	1 (ref.)		
Female	1.19	0.54–2.58	0.670
Tooth type			
Maxillary M1	1 (ref.)		0.104
Maxillary M2	4.13	0.96–17.7	0.057
Mandibular M1	5.11	1.37–19.1	0.015*
Mandibular M2	3.14	0.75–13.2	0.118
Proximal contacts			
Non‐missing	1 (ref.)		
Missing	1.05	0.25–4.40	0.948
Restoration			0.470
Temporary filling	1 (ref.)		
Crown	1.90	0.65–5.56	0.241
Core	1.13	0.10–13.4	0.921
Post			
No post	1 (ref.)		
Post	1.19	0.53–2.71	0.672
Apical terminus of RCF			
Adequate + Exact + Over	1 (ref.)		
Short	0.23	0.08–0.64	0.005**
Residual dentine (pericervical)			
≤ Traditional	1 (ref.)		
Excessive	2.40	0.99–5.78	0.051
Residual dentine (apical)			
≤ Traditional	1 (ref.)		
Excessive	13.0	5.33–31.8	< 0.001***
History of reRCT			
No	1		< 0.001***
1	5.71	1.75–18.6	0.004**
≥ 2	32.3	8.12–128.1	< 0.001***
Time from pRCT			
< 10 year	1 (ref.)		
≥ 10 year	2.46	0.99–6.10	0.053
Time from pRCT			
< 15 year	1 (ref.)		
≥ 15 year	6.46	2.64–15.8	< 0.001***

Abbreviations: M1, first molar; M2, second molar; pRCT, primary root canal treatment; RCF, root canal filling; reRCT, root canal re‐treatment.

* *p *< 0.05

** *p *< 0.01

*** *p *< 0.001.

### 3.2 Multiple Binary Logistic Regression Analysis

In this model, tooth type, residual apical dentine, a history of reRCTs, and time from pRCT ≥ 15 years were significant predictors of VRF (*p* < 0.05) (Table 4). The sensitivity, specificity, positive predictive value (PPV), and negative predictive value (NPV) were 85.7%, 89.5%, 78.9%, and 93.2%, respectively. ROC curve analysis revealed that this model was useful for discriminating VRFs from non‐VRFs in molars with an isolated PD ≥ 5 mm (AUC, 0.940 [0.899–0.981]; *p* < 0.001).

**Table 4:** Results of multiple binary logistic regression analysis. Adjusted odds ratio (OR) and 95% confidence interval (CI). Method stepwise to enter variables.

Variables	OR	95% CI	*p* value
Tooth type			
Maxillary M1	1 (ref.)		0.026*
Maxillary M2	2.59	0.19–34.9	0.474
Mandibular M1	19.2	2.44–150.5	0.005**
Mandibular M2	18.9	1.85–193.7	0.013*
Residual dentine (apical)			
≤ Traditional	1 (ref.)		
Excessive	12.9	2.92–57.5	0.001**
History of reRCT			
No	1 (ref.)		< 0.001***
1	9.89	1.68–58.2	0.011*
≥ 2	87.9	10.7–722.1	< 0.001***
Time from pRCT			
< 15 year	1 (ref.)		
≥ 15 year	20.7	4.20–102.0	< 0.001***

Abbreviations: M1, first molar; M2, second molar; pRCT, primary root canal treatment; reRCT, root canal re‐treatment.

* *p *< 0.05

** *p *< 0.01

*** *p *< 0.001.

### 3.3 Intra‐Observer and Inter‐Observer Reliability

The intra‐ and inter‐observer reliabilities for the apical terminus of the RCF and residual pericervical and apical dentine measurements were very good (> 0.8).

## 4 Discussion

VRFs are considered a consequence of fatigue from occlusal loading (Chan et al. 1999). This process involves stress concentration, crack initiation, crack propagation, and fracture (Kishen 2015). Root canal preparation, post placement, and compaction during obturation cause dentine loss (Ikram et al. 2009; Ossareh et al. 2018), and stress concentration in the root (Lertchirakarn et al. 2003; Kim et al. 2010; Rundquist and Versluis 2006), which may induce defects or cracks (Shemesh et al. 2009; Adorno et al. 2011), alter the stress distribution from occlusal loading, and create additional sites of stress concentration (Ossareh et al. 2018; Yoon et al. 2018; Yuan et al. 2018; Zhou et al. 2023), consequently, accelerating the occurrence of VRF. Previous ex‐vivo studies have investigated the effects of these factors; however, they cannot replicate the clinical situation. Clinical studies on this topic are scarce, and most studies are retrospective and focused on the presence of a post. Furthermore, the frequency of VRFs varies depending on the tooth type. To date, no study has focused exclusively on molars. The present study investigated the association between endodontic factors—particularly root dentine loss—and VRFs in ETMs.

Previous studies have reported a higher frequency of VRF in mandibular first molars (Lee et al. 2023; Chan et al. 1999; Yoshino et al. 2015; Guldener et al. 2017). In this study, the mandibular first molar was the most common tooth type in both VRF group (52.3%; 23/44) and controls (35.9%; 33/92) (Figure 3C), and was a significant risk factor for VRF in the multiple binary logistic regression model (OR, 19.2; 95% CI, 2.44–150.5, *p* = 0.005, Table 4). Among mandibular first molars in cases (52.3%; 23/44), VRF was prevalent in the mesial roots (69.6%; 16/23). This finding is in accordance with previous studies (Chan et al. 1999; Guldener et al. 2017). Chan et al. (1999) reported that the mesial roots of the mandibular first molars were the most susceptible to VRF, regardless of root canal treatment. Different stress distribution characteristics between mesial and distal roots were also reported (Yoon et al. 2018). Mandibular first molars may be particularly prone to VRF due to (para) functional loading, and mesial root morphology. When combined with endodontic or restorative factors, these teeth may become particularly susceptible to VRF. In contrast, premolars, which were frequent exclusively in VRF (Lee et al. 2023; Pan et al. 2022; Patel et al. 2025), might increase susceptibility to VRF solely due to iatrogenic factors such as post placement (Pan et al. 2022).

Previous studies reported significant association between posts and VRF (Lee et al. 2023; Maddalone et al. 2018; Fuss et al. 2001). However, when samples were limited to molars in this study, this factor was not significant (*p* = 0.672, Table 3). The contradictory results may be attributed to sample selection. In our previous study, two‐thirds of the teeth with posts in the VRF group were premolars and anterior teeth (Lee et al. 2023). Previous studies also included various tooth types, and the frequency distribution of teeth with posts according to the tooth type was unknown (Maddalone et al. 2018; Fuss et al. 2001). In this study, VRFs were most common in the mesial roots (61.4%; 27/44), with the majority not containing a post (24/27). This finding aligns with Guldener et al. (2017), who also reported VRFs most frequently in mesial roots of mandibular first molars without a post.

Previous studies reported higher survival rate of crowned ETT than non‐crowned ETT (Salehrabi and Rotstein 2004); however, this factor was not significant (*p* = 0.241, Table 3). In this study, the majority of cases and controls were crowned (Figure 3E). The result might be confounded by endodontic factors, since cuspal coverage restoration is recommended for ETMs. The effect of crown on VRF prevention/development has not been investigated. Further controlled prospective study is necessary to investigate this factor.

Root canal shaping/filling beyond the apex is considered a risk factor for VRF because it induces microcracks (Adorno et al. 2011; PradeepKumar et al. 2019). However, teeth rated as “over” were the least common (4.5%; 2/44) in cases (Figure 3G). Although teeth rated as “exact” based on PAs could be “over” in practice, the frequency of teeth with “exact” in the VRF group was 22.7% (10/44) and most VRFs had “adequate” apical terminus (61.4%; 27/44) (Figure 3G). Teeth rated as “short” were 35.9% (33/92) in controls and 11.3% (5/44) in cases and “short” category decreased the probability of VRF in the simple binary logistic regression model (*p* = 0.005, Table 3). The result might be attributed to differences in the iatrogenic factors that the teeth had received. From the PAs, it is inferred that the “short” apical terminus of RCF was caused by insufficient root canal preparation due to treatment difficulties, such as calcified or curved root canals and ledge formation. Poor‐quality RCF can also lead to “short” apical terminus of RCF. Therefore, these teeth likely experienced fewer iatrogenic factors—such as mechanical stress from root canal preparation, apical dentine loss, and compaction force during RCF—compared to those in the “adequate”, “exact”, and “over” categories.

Root canal treatment results in pericervical and apical dentine loss, which, if excessive, contributes to VRF (Ossareh et al. 2018; Yuan et al. 2018). Silva et al. (2021) calculated the mean dentine thickness of root filled teeth with VRF and non‐VRF using CBCT images and reported that the remaining dentine thickness was significantly associated with VRF. In their study, pre‐assessment calibration and intra‐examiner agreement were not confirmed and CBCT images with the voxel sizes of 0.125–0.2 mm, FOV 16 × 4 cm were used. Furthermore, they used arithmetic mean dentine thickness (cervical, middle, apical) of teeth with VRF for the assessment and single‐ and multi‐rooted teeth were mixed. In the present study, residual pericervical and apical dentine volume were assessed individually in one view using PAs and cut‐off values were established based on references (intact root canals and root canals with minimum/traditional preparations) (Table 1). Although assessment in three‐orthogonal views with CBCT could be more informative, we felt that PA assessment was more clinically relevant as most general practitioners are familiar with PAs and therefore can use this radiographic assessment to determine the likelihood and/or risk factors for VRF and advise their patients accordingly. However, PA do have limitations, these include the two‐dimensional assessment may cause distortion of the image, which could affect the measured dentine thickness. Furthermore, PA does not measure the proximal thickness of roots in the buccal to lingual plane.

Although the importance of preserving pericervical dentine has been advocated (Clark and Khademi 2010), residual pericervical dentine was not significant in this study, while apical dentine was significant in the multiple logistic regression models (Table 4). This may be due to difference of original dentine volume. Additionally, apical preparation may cause greater stress concentration than cervical preparation because of the smaller canal diameter and may create defects or microcracks (Adorno et al. 2011). Moreover, greater apical preparation requires multiple steps that may exacerbate the mechanical stress to the root (Jamleh et al. 2021). Thus, excessive apical root preparation can make the residual apical dentine more prone to VRF. The impact of residual apical dentine on VRF was also supported by “apical terminus of RCF” in which “Short” category decreased the probability of VRF (OR, 0.23; 95% CI, 0.08–0.64, *p* < 0.005, Table 3).

Microcracks are considered as risk factors for VRF because they can be sites of stress concentration. However, the results of previous studies on microcrack formation after root canal preparation and filling are inconsistent (Shemesh et al. 2009; Adorno et al. 2011; De‐Deus et al. 2014) and a recent review article on this topic concluded that dentinal microcracks are a laboratorial phenomenon (Versiani et al. 2022).

Microcrack formation may vary according to the operator experience and preoperative condition of root (root filled/non‐root filled, curvature/non‐curvature, wide/obliterated). These factors are non‐measurable in laboratory studies.

A higher stress is produced in areas with flaws or defects (stress concentration), and the material fractures easily with small loads (Kishen 2015; Kinney et al. 2003). Therefore, a fracture may start at any part of the root with stress concentration (e.g., cracks, defects, or curvature). VRF development and the time required for progress may depend on residual dentine thickness, structural properties, and occlusal loading factors (direction, location, and magnitude of the force) (Chan et al. 1999). Therefore, even non‐ETT can develop VRF over time.

ReRCT causes additional dentine loss and mechanical stress to the root. Therefore, it may fasten VRF development (Ganesh et al. 2014). History of previous reRCT was a strong risk factor for VRF in the multiple binary logistic regression analysis (OR, 87.9; 95% CI, 10.7–722.1, *p* < 0.001) (Table 4). Among cases, 89.7% (35/39) had history of reRCT and 46.1% (18/39) had a history of two or more reRCTs (Figure 3J).

The probability of VRF significantly increased when the time elapsed since pRCT was ≥ 15 years (OR, 20.7; 95% CI, 4.20–102.0, *p* < 0.001) (Table 4). ETT might experience fatigue earlier depending on the residual dentine volume and the cumulative damage to the root. In previous studies (PradeepKumar et al. 2016; Fuss et al. 2001; Testori et al. 1993), the time elapsed before VRF ranged from 4.35 ± 1.96 years (PradeepKumar et al. 2016) to 10 years (Testori et al. 1993). These results are inconsistent with those of the present study, which could be attributed to the heterogeneities in previous studies. Fuss et al. (2001) included teeth that underwent both pRCTs and reRCTs, whereas PradeepKumar et al. (2016) included only teeth that underwent pRCTs, and the other lacked the information (Testori et al. 1993). Attrition bias, which hinders the identification of the true effects, cannot be ruled out in this study. A more controlled time‐course analysis is necessary to clarify the accurate weight of this factor on VRF.

## 5 Limitations of the Present Study

1. Study design
  The retrospective observational study has inherent limitations. Ideally, cases and controls should be matched based on relevant variables. However, due to sample size constraints, matching was only possible for tooth type (molars) and root canal treatment status (root‐filled teeth).
2. Unaccounted factors potentially contributing to VRFs
  Several potentially important contributory factors to VRFs were not assessed. These include parafunctional habits, preoperative dentine volume/canal sizes, coronal dentine loss above the canal orifices, and axial root morphology. Data on instrumentation techniques, file systems, and RCF techniques were also unavailable due to the retrospective nature of the study. All patients had previously received RCT at general dental practices in Japan, where hand files and the cold lateral condensation technique are commonly used. It is therefore reasonable to assume that these techniques were used. These procedural factors should be considered when interpreting the findings.
3. Limitations of two‐dimensional assessment
  Two‐dimensional radiographic assessment may cause image distortion, which could affect the measurement of dentine thickness. Furthermore, it was not feasible to assess the remaining root dentine thickness in the bucco‐lingual dimension.
4. Potential confounding factors
  Due to heterogeneity in several uncontrolled variables, confounding may have influenced the results. To accurately determine the effect of remaining root dentine thickness on VRF development, these factors should be carefully controlled. Such control is only feasible in well‐designed prospective studies. Therefore, the findings of the present study should be interpreted with caution.
5. Sample characteristics
  All patients were Japanese, which may limit generalizability to other populations (Lee et al. 2023). In Japan, endodontic specialist practices are uncommon, and the national health insurance system offers inexpensive root canal treatment. Consequently, patients are more likely to attempt to retain their teeth rather than opt for extraction. This tendency is evidenced by 46.1% of cases having a history of two or more reRCTs (Figure 3J). These factors may contribute to the high prevalence of VRF in the Japanese population (Lee et al. 2023; Yoshino et al. 2015).

## 6 Conclusion

Within the limitations of this study, residual apical dentine had a stronger correlation with VRF than pericervical dentine. A history of reRCT significantly increased the risk of VRF. In ETMs with an isolated PD ≥ 5 mm, the residual apical dentine, tooth type, history of reRCT, and time from pRCT ≥ 15 years may be useful parameters to identify teeth with VRF (AUC, 0.940; PPV, 78.9%; NPV, 93.2%; *p* < 0.001). Successful pRCT with minimum canal preparation, particularly at the apical level, is critical to preventing VRF, especially in teeth predisposed to VRF, such as mandibular first molars.

## Author Contributions

**Kwangsoon Lee:** conceptualization, methodology, investigation, resources, writing – original draft, writing – review and editing. **Shanon Patel:** writing – review and editing, supervision. **Manjeet Ahlowalia:** writing – review and editing. **Ruth Perez Alfayate:** writing – review and editing. **Federico Foschi:** review and editing, supervision.

## Funding

The authors received no specific funding for this work.

## Ethics Statement

This study was approved by the BDM research ethics office of King's College London (LRU‐20/21‐21498 A).

## Consent

Written consent was obtained from the patients through a consent for the use of patient's clinical data for the study.

## Conflicts of Interest

The authors declare no conflicts of interest.

## Supporting information

Supplementary_tables_T1–T12.

## Data Availability

The data that support the findings of this study are openly available upon request.
