# Supplementary material for: Association Between Residual Pericervical and Apical Dentine and Vertical Root Fracture in Endodontically Treated Molars: A Case‐Control Study
Source: Clin Exp Dent Res. 2026 Feb 3;12(1):e70293. doi: 10.1002/cre2.70293 (PMC12867462; doi:10.1002/cre2.70293)
Supplement: Supplementary file 1 — Supplementary_tables_T1–T12. [file CRE2-12-e70293-s001.docx]

**Supplementary tables (T1–T12)**

The frequency distribution of 12 variables in cases and controls is shown in T1-T12.

**T1.** Age

|  | **AGE GROUP** | | | | | | | | | |
| --- | --- | --- | --- | --- | --- | --- | --- | --- | --- | --- |
|  | **Total** | | **<40 y** | | **40-49 y** | | **50-59 y** | | **>=60 y** | |
|  | **N** | **%** | **N** | **%** | **N** | **%** | **N** | **%** | **N** | **%** |
| **Total** | 136 | 100.0 | 37 | 27.2 | 43 | 31.6 | 38 | 27.9 | 18 | 13.2 |
| **VRF** | 44 | 100.0 | 12 | 27.3 | 15 | 34.1 | 11 | 25.0 | 6 | 13.6 |
| **Non-VRF** | 92 | 100.0 | 25 | 27.2 | 28 | 30.4 | 27 | 29.3 | 12 | 13.0 |

**T2.** Sex

|  | **SEX** | | | | | |
| --- | --- | --- | --- | --- | --- | --- |
|  | **Total** | | **male** | | **female** | |
|  | **N** | **%** | **N** | **%** | **N** | **%** |
| **Total** | 136 | 100.0 | 40 | 29.4 | 96 | 70.6 |
| **VRF** | 44 | 100.0 | 14 | 31.8 | 30 | 68.2 |
| **Non-VRF** | 92 | 100.0 | 26 | 28.3 | 66 | 71.7 |

**T3.** Tooth type

|  | **TOOTH TYPE** | | | | | | | | | |
| --- | --- | --- | --- | --- | --- | --- | --- | --- | --- | --- |
|  | **Total** | | **upper M1** | | **upper M2** | | **lower M1** | | **lower M2** | |
|  | **N** | **%** | **N** | **%** | **N** | **%** | **N** | **%** | **N** | **%** |
| **Total** | 136 | 100.0 | 25 | 18.4 | 25 | 18.4 | 56 | 41.2 | 30 | 22.1 |
| **VRF** | 44 | 100.0 | 3 | 6.8 | 9 | 20.5 | 23 | 52.3 | 9 | 20.5 |
| **Non-VRF** | 92 | 100.0 | 22 | 23.9 | 16 | 17.4 | 33 | 35.9 | 21 | 22.8 |

**T4.** Proximal contacts

|  | **PROXIMAL CONTACTS** | | | | | |
| --- | --- | --- | --- | --- | --- | --- |
|  | **Total** | | **Missing** | | **Non-missing** | |
|  | **N** | **%** | **N** | **%** | **N** | **%** |
| **Total** | 136 | 100.0 | 9 | 6.6 | 127 | 93.4 |
| **VRF** | 44 | 100.0 | 3 | 6.8 | 41 | 93.2 |
| **Non-VRF** | 92 | 100.0 | 6 | 6.5 | 86 | 93.5 |

**T5.** Restoration

|  | **RESTORATION** | | | | | | | | | |
| --- | --- | --- | --- | --- | --- | --- | --- | --- | --- | --- |
|  | **Total** | | **Crown** | | **Core** | | **Temporary**  **filling** | | **CR, In, On** | |
|  | **N** | **%** | **N** | **%** | **N** | **%** | **N** | **%** | **N** | **%** |
| **Total** | 136 | 100.0 | 106 | 77.9 | 4 | 2.9 | 22 | 16.2 | 4 | 2.9 |
| **VRF** | 44 | 100.0 | 38 | 86.4 | 1 | 2.3 | 5 | 11.4 | 0 | .0 |
| **Non-VRF** | 92 | 100.0 | 68 | 73.9 | 3 | 3.3 | 17 | 18.5 | 4 | 4.3 |

**T6.** Post

|  | **POST** | | | | | |
| --- | --- | --- | --- | --- | --- | --- |
|  | **Total** | | **no** | | **yes** | |
|  | **N** | **%** | **N** | **%** | **N** | **%** |
| **Total** | 136 | 100.0 | 102 | 75.0 | 34 | 25.0 |
| **VRF** | 44 | 100.0 | 32 | 72.7 | 12 | 27.3 |
| **Non-VRF** | 92 | 100.0 | 70 | 76.1 | 22 | 23.9 |

**T7.** Apical terminus of RCF

|  | **Apical terminus of RCF** | | | | | | | | | |
| --- | --- | --- | --- | --- | --- | --- | --- | --- | --- | --- |
|  | **Total** | | **Short** | | **Adequate** | | **Exact** | | **Over** | |
|  | **N** | **%** | **N** | **%** | **N** | **%** | **N** | **%** | **N** | **%** |
| **Total** | 136 | 100.0 | 38 | 27.9 | 75 | 55.1 | 21 | 15.4 | 2 | 1.5 |
| **VRF** | 44 | 100.0 | 5 | 11.4 | 27 | 61.4 | 10 | 22.7 | 2 | 4.5 |
| **Non-VRF** | 92 | 100.0 | 33 | 35.9 | 48 | 52.1 | 11 | 12.0 | 0 | 0.0 |

**T8.** Residual pericervical dentin

|  | **Residual pericervical dentin** | | | | | | | |
| --- | --- | --- | --- | --- | --- | --- | --- | --- |
|  | **Total** | | **Minimum** | | **Traditional** | | **Excessive** | |
|  | **N** | **%** | **N** | **%** | **N** | **%** | **N** | **%** |
| **Total** | 136 | 100.0 | 28 | 20.6 | 12 | 8.8 | 96 | 70.6 |
| **VRF** | 44 | 100.0 | 3 | 6.8 | 5 | 11.4 | 36 | 81.8 |
| **Non-VRF** | 92 | 100.0 | 25 | 27.2 | 7 | 7.6 | 60 | 65.2 |

**T9.** Residual apical dentin

|  | **Residual apical dentin** | | | | | | | |
| --- | --- | --- | --- | --- | --- | --- | --- | --- |
|  | **Total** | | **Minimum** | | **Traditional** | | **Excessive** | |
|  | **N** | **%** | **N** | **%** | **N** | **%** | **N** | **%** |
| **Total** | 136 | 100.0 | 74 | 54.4 | 25 | 18.4 | 37 | 27.2 |
| **VRF** | 44 | 100.0 | 12 | 27.2 | 5 | 11.4 | 27 | 61.4 |
| **Non-VRF** | 92 | 100.0 | 62 | 67.4 | 20 | 21.7 | 10 | 10.9 |

**T10.** History of reRCT

|  | **History of reRCT** | | | | | | | |
| --- | --- | --- | --- | --- | --- | --- | --- | --- |
|  | **Total** | | **No** | | **1** | | **2 or more** | |
|  | **N** | **%** | **N** | **%** | **N** | **%** | **N** | **%** |
| **Total** | 120 | 100.0 | 47 | 39.2 | 49 | 40.8 | 24 | 20.0 |
| **VRF** | 39 | 100.0 | 4 | 10.3 | 17 | 43.6 | 18 | 46.1 |
| **Non-VRF** | 81 | 100.0 | 43 | 53.1 | 32 | 39.5 | 6 | 7.4 |

**T11.** Time from pRCT (1)

|  | **Time from pRCT (1)** | | | | | |
| --- | --- | --- | --- | --- | --- | --- |
|  | **Total** | | **Less than 10Y** | | **10Y or more** | |
|  | **N** | **%** | **N** | **%** | **N** | **%** |
| **Total** | 111 | 100.0 | 40 | 36.0 | 71 | 64.0 |
| **VRF** | 35 | 100.0 | 8 | 22.9 | 27 | 77.1 |
| **Non-VRF** | 76 | 100.0 | 32 | 42.1 | 44 | 57.9 |

**T12.** Time from pRCT (2)

|  | **Time from pRCT (2)** | | | | | |
| --- | --- | --- | --- | --- | --- | --- |
|  | **Total** | | **Less than 15Y** | | **15Y or more** | |
|  | **N** | **%** | **N** | **%** | **N** | **%** |
| **Total** | 111 | 100.0 | 78 | 70.3 | 33 | 29.7 |
| **VRF** | 35 | 100.0 | 15 | 42.9 | 20 | 57.1 |
| **Non-VRF** | 76 | 100.0 | 63 | 82.9 | 13 | 17.1 |
